# Physical Activity Modulates miRNAs Levels and Enhances MYOD Expression in Myoblasts

**DOI:** 10.1007/s12015-022-10361-9

**Published:** 2022-03-22

**Authors:** Luca Dalle Carbonare, Gianluigi Dorelli, Veronica Li Vigni, Arianna Minoia, Jessica Bertacco, Samuele Cheri, Michela Deiana, Giulio Innamorati, Mattia Cominacini, Cantor Tarperi, Federico Schena, Monica Mottes, Maria Teresa Valenti

**Affiliations:** 1 Department of Medicine, University of Verona, 37100 Verona, Italy; 2 Department of Neurosciences, Biomedicine and Movement Sciences, University of Verona, 37100 Verona, Italy

**Keywords:** MicroRNAs, Half Marathon, Myogenesis, MYOD

## Abstract

Stem cells functions are regulated by different factors and non-conding RNAs, such as microRNA. MiRNAsplay an important role in modulating the expression of genes involved in the commitment and differentiation of progenitor cells. MiRNAs are post transcriptional regulators which may be modulated by physical exercise. MiRNAs, by regulating different signaling pathways, play an important role in myogenesis as well as in muscle activity. MiRNAs quantification may be considered for evaluating physical performance or muscle recovery. With the aim to identify specific miRNAs potentially involved in myogenesis and modulated by physical activity, we investigated miRNAs expression following physical performance in Peripheral Blood Mononuclear Cells (PBMCs) and in sera of half marathon (HM) runnners. The effect of runners sera on Myogenesis in in vitro cellular models was also explored. Therefore, we performed Microarray Analysis and Real Time PCR assays, as well as in vitro cell cultures analysis to investigate myogenic differentiation. Our data demonstrated gender-specific expression patterns of PBMC miRNAs before physical performance. In particular, miR223-3p, miR26b-5p, miR150-5p and miR15-5p expression was higher, while miR7a-5p and miR7i-5p expression was lower in females compared to males. After HM, miR152-3p, miR143-3p, miR27a-3p levels increased while miR30b-3p decreased in both females and males: circulating miRNAs mirrored these modulations. Furthermore, we also observed that the addition of post-HM participants sera to cell cultures exerted a positive effect in stimulating myogenesis. In conclusion, our data suggest that physical activity induces the modulation of myogenesis-associated miRNAs in bothfemales and males, despite the gender-associated different expression of certain miRNAs, Noteworthy, these findings might be useful for evaluating potential targets for microRNA based-therapies in diseases affecting the myogenic stem cells population.

**Graphical Abstract:**

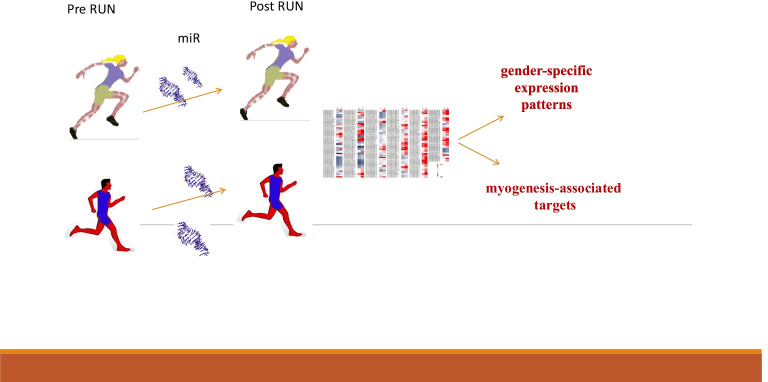

**Supplementary Information:**

The online version contains supplementary material available at 10.1007/s12015-022-10361-9.

## Introduction

Specific miRNA signatures either allow stemness e maintenance or support stem cells differentiation [1]. However, miRNAs can also affect stem cells activity; their involvement in the dysregulation of stem cells has been demonstrated in lung injury, in Systemic Sclerosis, Ischemic Myocardium, in osteoporosis and in osteopenia as well as in Autism Spectrum Disorder [2]. In the complex scenario of gene expression regulation, micro RNAs (miRNAs) play an important role by targeting specific genes at the post-transcriptional level. In addition, microRNA based-therapies have been exploited as potential treatments for traumatic or degenerative diseases [3, 4]. MiRNAs regulation is also involved in myogenesis and muscle stem cell (MuSC) ageing as well as in the osteogenic commitment of MSCs in health and disease [5, 6]. MiRNAs have been identified as cell signaling mediators after cellular stress [7]. In mammals, miRNAs have been associated to the regulation of > 50% protein-coding genes expression [8].The molecular mechanisms involved in body adaptation following physical activity have not been clarified yet. However, it has been suggested that physical activity is able to modulate the expression of various genes by inducing or reducing the expression of different miRNAs [9, 10].

In the last years, running has been gaining increasing attractiveness due to its positive effects on overall health. A constantly growing number of recreational runners is taking part mainly in 10 km and half-marathon races, where the physical effort is lower than in longer distance races (such as marathon); yet the health benefits of endurance exercise are appreciable [11].

Body adaptation to exercise depends on the modulated expression of genes coding for proteins associated to physical activity [12, 13]. The exact molecular mechanisms responsible for body adaptation to exercise have not been defined.

During a half marathon race, recreational runners usually sustain an exercise intensity between 75% and 85% of the maximal aerobic capacity (VO2max) for about 80 to 120 min [11, 14]. Many acute cellular responses are detectable, e.g. an increase in mitochondrial oxidative phosphorylation, which in turn raises the production of reactive oxygen species (ROS) [15]. Furthermore, an increased anoxic regeneration of the oxidized form of nicotinamide adenine dinucleotide (NAD+) during glycolysis, produces lactates and H+ [16].

All these processes are responsible for muscle fatigue and exercise-induced muscle injury [7, 8]. They underlie chronic training adaptations and the positive effects of endurance exercise, which include cardiomyogenesis, optimization of peripheral tissue microcirculation, muscular satellite cells increase and modulation of mesenchymal cells differentiation [17]. In this context, miRNAs expression is enhanced under injury conditions or for excessive stress [18]. In fact, miRNAs can be considered modulators of inflammation and mitochondrial metabolism; they are involved in muscle recovery and hypertrophy [19]. Myogenesis and muscle activity are regulated by different signaling pathways which in turn are modulated by a large spectrum of miRNAs. In particular, both ubiquitous miRNAs and tissue-specific miRNAs are involved during muscle differentiation and activity [20].

Therefore, miRNAs expression modulation plays an important role during physical exercise; varying miRNA levels can be considered useful biomarkers for evaluating physical performance or stress, as well as muscle recovery.

## Materials and Methods

### Participants

38 age-matched runners (18 women and 20 men) carried out a 21.1 km half marathon. Study participants were enrolled during the sport event called “run for science,” held in Verona (Italy) in April 2019. The participants (median age: women 41.4 ± 7.2; men 41.7 ± 8.3) were all physically fit recreational runners. Bone Mineral Index (BMI) and CTX value were: median BMI (women 21.6 ± 1.3; men 23.1 ± 1.8); median CTX (women 0.34 ± 0.08 mg/ml; men 0.36 ± 0.03 mg/ml). All runners underwent a clinical evaluation as well as a medical history interview to exclude comorbidities or drugs intake. Written informed consent was obtained from all participants, and the study was approved by the ethical committee of Azienda Ospedaliera Universitaria Integrata of Verona, Italy (number 1538; Dec. 3, 2012; local ethical committee of Azienda Ospedaliera Integrata di Verona). The study design and methods comply with the Declaration of Helsinki.

### Peripheral Blood Mononuclear Cells (PBMCs) and Sera Collection

Peripheral blood was collected by venipuncture before the run and immediately after, upon informed consent. Mononuclear cells (PBMCs) and sera were obtained with standard procedures as previously reported 12. Then, PBMCs and sera were stored at − 80 ◦C until use.

### In Vitro Myogenic Differentiation

Human Skeletal muscle cells (SKMC) were purchased from PromoCells (C-12,580; PromoCell, GMBH Heidelberg, Germany) and cultured with Skeletal Muscle Cell Growth medium (Low serum) (C-23060PromoCell, GMBH Heidelberg, Germany) as recommended by manufacturer’s instructions., SKMCs were differentiated by using Skeletal Muscle Differentiation Medium Supplements (C-23,061; PromoCell, GMBH Heidelberg, Germany) with or without pooled sera of partecipants at 5% final concentration. The medium was changed every 3 days after initial plating.

### RNAs Extraction and Reverse Transcription

miRNAs were extracted from PBMCs by using miRNeasy kit (Qiagen, Hilden, Germany) accordingly to manufacturer’s instruction. Circulating miRNAs were exctracted from serum samples with the miRNeasy Serum/Plasma Advanced Kit (Qiagen, Hilden, Germany) according to the manufacturer’s instructions. [21]. Total RNA from differentiating SKMCs treated or untreated with pooled sera was exctracted with a “RNeasy® protect mini kit” (Qiagen, Hilden, Germany), following the manufacturer’s protocol. miRNAs or RNA samples were quantified by a Qubit™ 3 fluorometer using a “Qubit™ RNA HS assay kit” (Invitrogen, Carlsbad, USA). Two micrograms of the extracted RNAs were reverse transcribed with a TaqMan microRNA Reverse Transcription kit (Thermofisher Corporation, Waltham, MA, USA) or First Strand cDNA Synthesis kit (GE Healthcare, Little Chalfont, UK), as previously reported. RNA and cDNA samples were stored at -80 °C.

### PCR Array and Real Time RT-PCR

MiRNAs expression profiling was performed using TaqMan Advanced miRNA Human A Card (A34714; ThermoFisher Scientific) according to manufacturer’s instruction. The assay was carried out by using ViiA™7 Real-Time PCR System (Applied Biosystems). Four TaqMan Advanced miRNA Assay endogenous controls included in the card were used to data normalization and the ∆∆Ct method was used to evaluate the fold change as previously reported [22]. For mRNA or miRNAs expression analysis or array validation we used Real-time PCR using TaqMan Universal PCR Master Mix (Thermofisher Corporation, Waltham, MA, USA) and TaqMan pre-designed probes for each gene (miR 22-3p, 000398; miR 152-3p, 000475; miR 100 5p, 000437; miR 143-3p, 002249; miR 216-5p, 002220; miR 27a-3p 000408; miR 30b-5p, 000602; miR 200b-3p, 002251; U6 snRNA, 001973; MYOD, Hs00159528_m1; MYH2, Hs00430042_m1; ACTB, Hs99999903_m1).

### Immunofluorescence

Immunofluorescence analyses were performed as previously reported [23]. Briefly, cells were fixed and processed according to the manufacturer’s protocols. The Primary antibody MYOD (cat. #MA1-41017; Thermofisher) was diluted (as reported in the datasheet) in antibody dilution buffer and incubated overnight at 4 °C. Slides were then incubated with secondary antibodies goat mouse fluorescein conjugated (cat. Ap124f, Millipore, Burlington, Massachusetts, USA). Nuclear staining was performed by ProLong™ Gold Antifade Mountant with DAPI. Images were recorded using a Leica (Wetzlar, Germany) inverted microscope at 10x. To express data in a semiquantitative way, four different fields were measured for each sample, in three independent experiments with about 80–100 total cells.

### Western Blotting

Proteins were extracted by using Ripa buffer (Thermo Fisher Scientific, Waltham, MA,USA) and concentrations were calculated with BCA assay (Thermo Scientific, Waltham, MA, USA) as previously reported [22]. Protein samples were diluted in Laemmli’s sample buffer (Biorad, CA, US), heated for 5 min at 95 ◦C, and separated by sodium dodecyl sulfate − polyacrylamide gel electrophoresis (SDS PAGE), followed by transfer onto polyvinylidene difluoride (PVDF) membranes (Thermo Fisher Scientific, Waltham, MA, USA). PVDF membranes were probed with the primary (antiMyoD (MA1-41017; Thermo Scientific, Waltham, MA, USA), β ACTIN (BA3R; Thermo Scientific, Waltham, MA, USA); and secondary antibodies ( Anti-mouse (Cell Signaling, 7076). Signals were detected with a chemiluminescence reagent (ECL, Millipore, Burlington, MA, USA) and Images were recorded using a LAS4000 Digital Image Scanning System (GE Healthcare, Little Chalfont, UK). Densitometric analysis was performed as we previously reported [24].

### Statistical Analysis

Results were expressed as mean ± SD. Statistical analysis was assessed by two-tailed Student’s paired-test. Differences were considered positive when p < 0.05. For in vitro data, analyses were applied to experiments carried out at least three times. We used SPSS for Windows, version 22.0 (SPSS Inc., Chicago, IL, USA) to analyse the data.

## Results

In order to identify potential miRNAs modulation following HM, at first we evaluated microRNAs expression by array analysis in PBMCs collected before in male and female before HM. This initial evaluation was performed to explore potential difference gender associated. Then, we performed array analysis in PBMCs collected after HM and, by comparing miRNAs profiles of PBMCs collected before and after HM, we evaluated miRNAs modulation following physical activity. The modulated miRNAs identified in PBMCs were also tested in sera to evaluate the modulation of circulating miRNAs following HM. Therefore, we investigated the effects of runners sera participants on myogenesis by performing in vitro experiments.

## Gender Associated microRNAs Profiles in Peripheral Blood

**Fig. 1:**
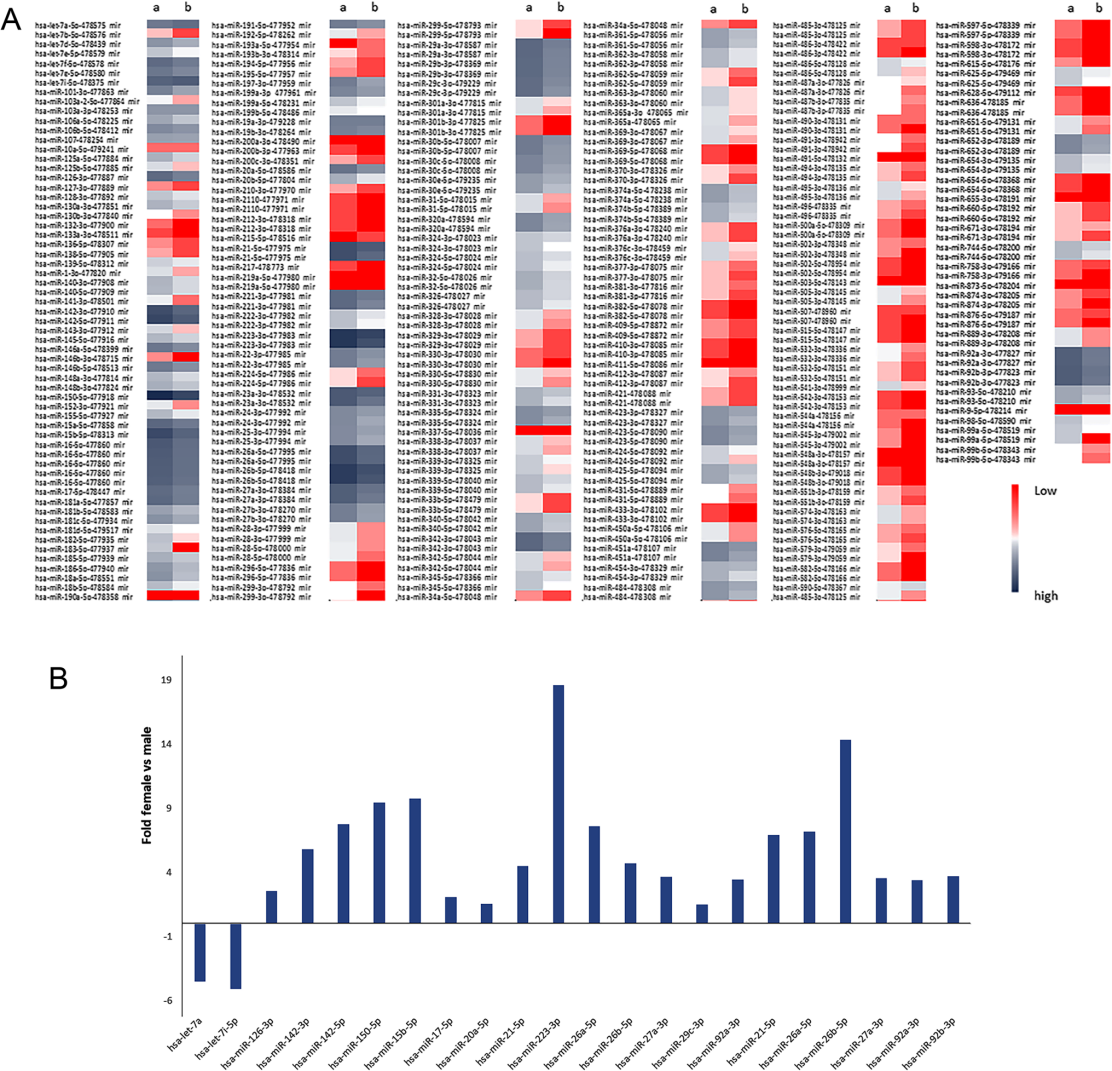
miRNAs array profiles in PBMCs in females (**a**) and males (**b**) (**A**). By comparing the array profiles, gender-related different miRNAs expression was observed in PBMCs. As shown in the graph (**B**), female PBMCs expressed higher levels of different miRNAs compared to male PBMCs. On the contrary, miRNAs 7a and 7i expression was lower in female PBMCs compared to male PBMCs

We analysed microRNAs expression in mononuclear cells collected from peripheral blood of age-matched females and males (18 women and 20 men). The array profiles analysed in females and males (Fig. 1A, Tables 1S and 2S) showed different expression profiles only for some miRNAs (Fig. 1B). Therefore, we observed some gender-associated PBMC miRNAs profiles. In detail:, miR223-3p. miR26b-5p, miR150-5p and miR15-5p expression was higher, while miR7a-5p and miR7i-5p expression was lower in females compared to males. In particular, miR223 expression was > 17x and miR26b > 14x in the female/male comparison.

### Half Marathon Modulates Similarly the Expression of Other PBMCs miRNAs Regardless of Gender

**Fig. 2:**
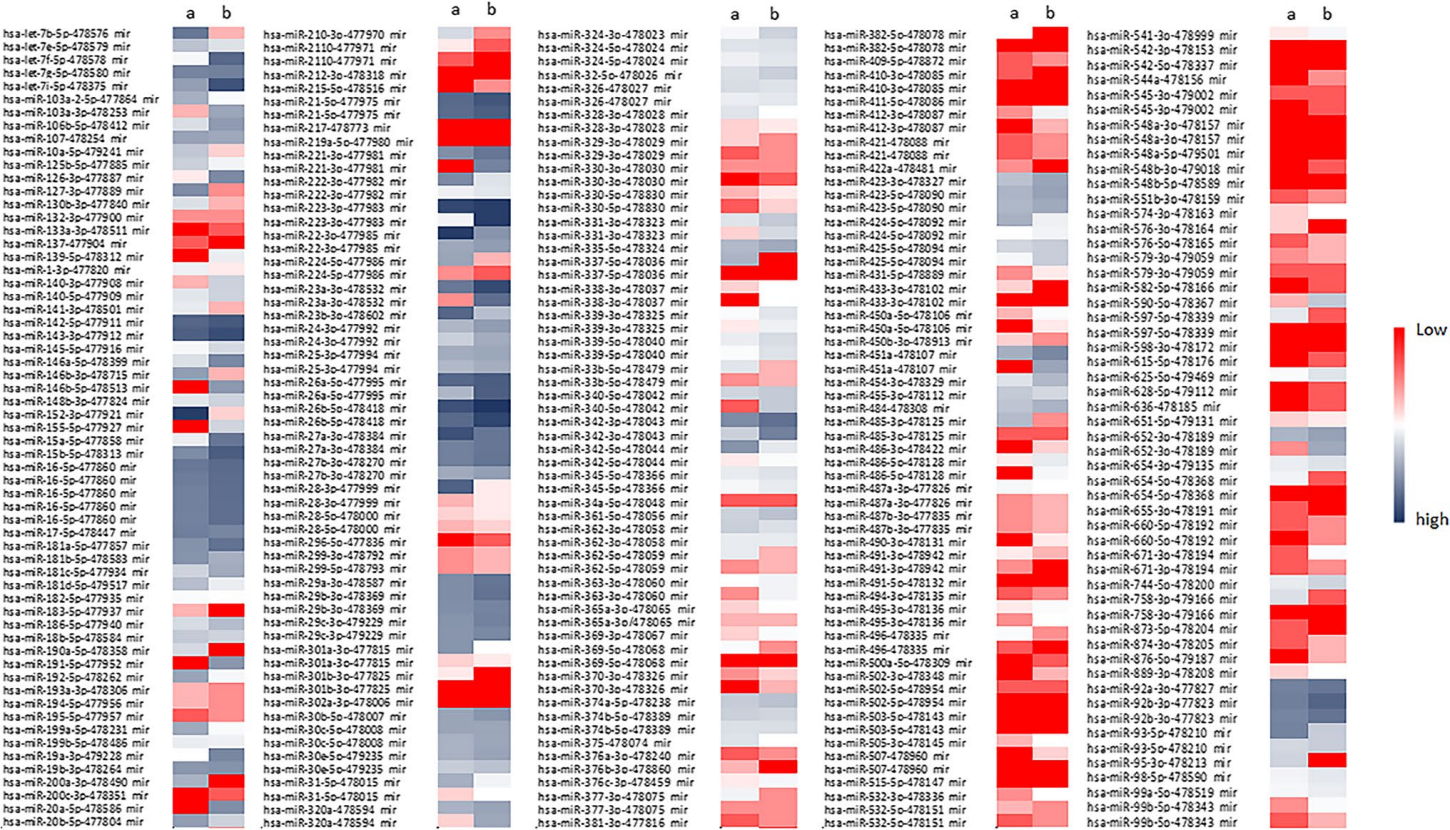
miRNAs array profiles in female -PBMCs (**A**) and male-PBMCs (**B**) after half marathon (HM)

After physical activity, we analysed miRNAs profiles in PBMCs of both females and males (Fig. 2; Tables 3S and 4S).

**Fig. 3:**
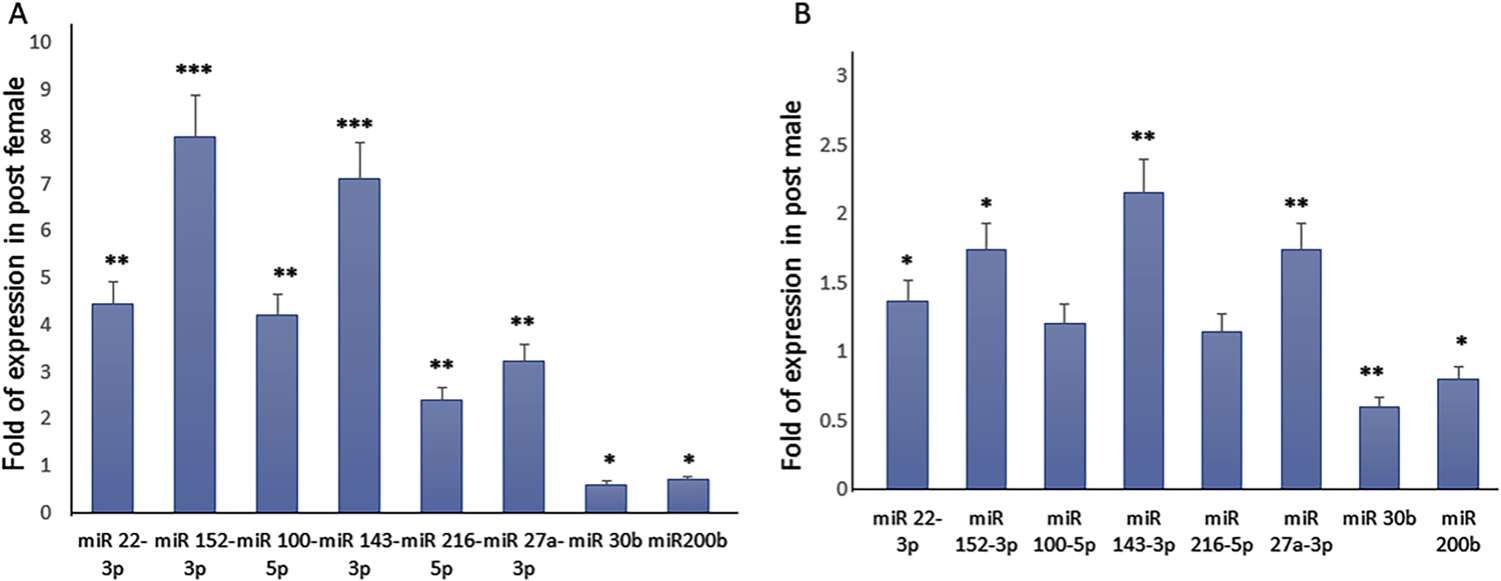
miRNAs modulation in female-PBMCs (**A**) and male-PBMCs (**B**). We observed in both female and male PBMCs and increased expression of miR152-3p, miR143-3p, miR27a-3p and a reduced expression of miR30b-5p and miR200b-3p after the HM compared to the expression before HM. However, the increased expression of miR 22-3p, miR100-5p and miR216-5p was observed only in female-PBMCs. Data are shown as mean ± standard deviation (SD); comparison was performed versus pre HM in female or in male. *p < 0.05; **p < 0.005; ***p < 0.001

By comparing the expression profiles, we observed similar miRNAs modulation in female and male PBMCs for some miRNAs, which did not appear to be gender-associated. In particular, we observed an increased expression of miR152-3p, miR143-3p, miR27a-3p, and a reduced expression of miR30b-3p after the half marathon (Fig. 2). However, we also observed an increased expression of miR 22-3p, miR100-5p, and miR216-5p in females post half marathon (pHM)-PBMCs but not in males. Real Time PCR gene expression analysis for some miRNAs confirmed array results (Fig. 3).

### Modulation of Circulating miRNAs After Half Marathon

**Fig. 4:**
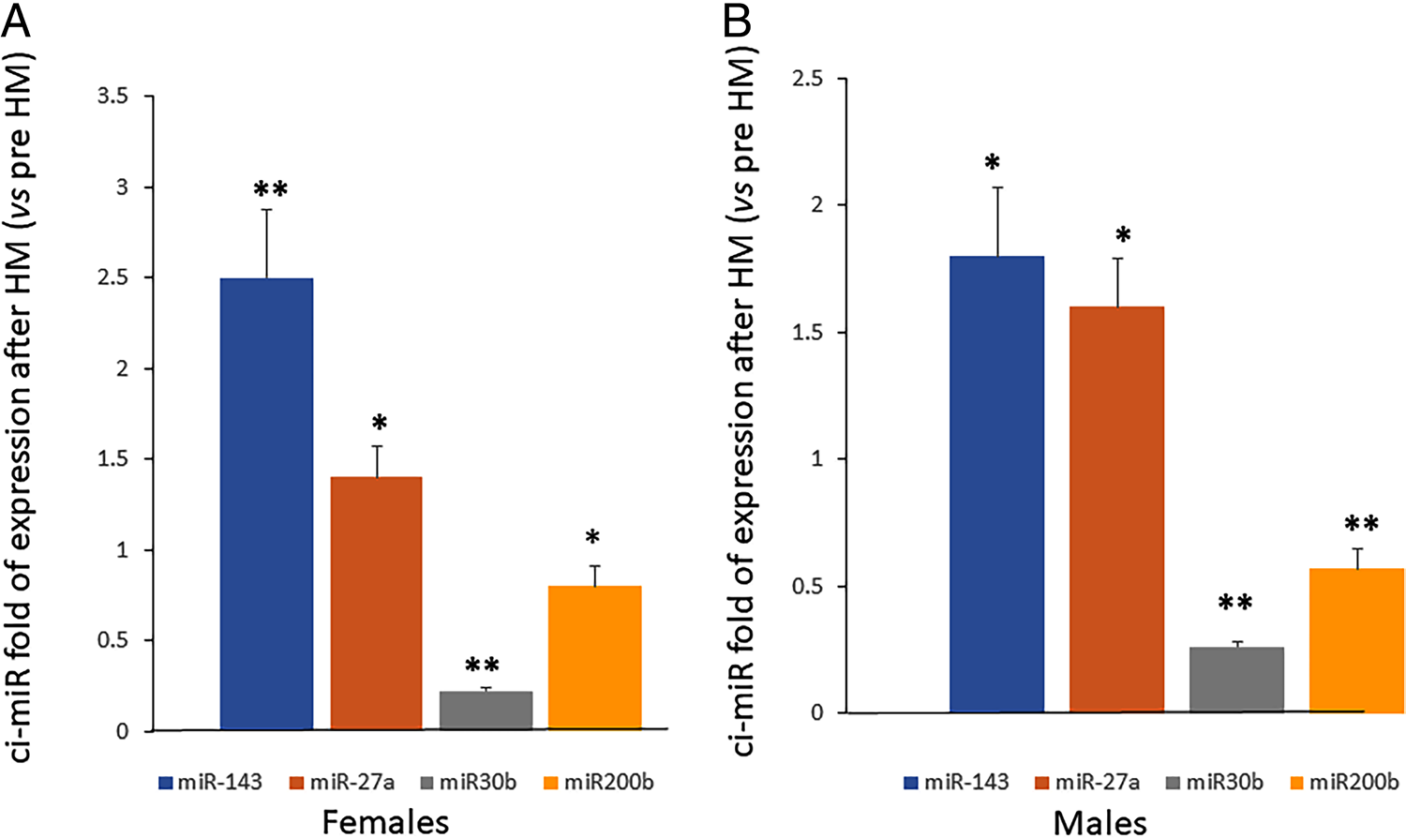
Increased expression of circulating miRNAs 143-3p and 27a-3p and reduced expression of miR30b-5p, miR200b-3p, following HM was observed in both female (**A**) and male (**B**) sera. Data are shown as mean ± standard deviation (SD); comparison was performed versus pre HM in female or in male. *p < 0.05; **p < 0.005

For some of the above-mentioned miRNAs, we evaluated the corresponding expression levels in circle, by collecting sera before and after the performance. We observed an increased expression of circulating miRNAs 143-3p and 27a-3p and a reduced expression of miR30b-5p and miR200b-3p following HM, in agreement with the observations in PBMCs (Fig. 4).

### Half Marathon Performance Enhances MYOD Expression in Myoblasts

As post HM myogenesis-related circulating miRNAs appeared to be modulated, we investigated the effects of runners’ sera on cultured cells during myogenic differentiation. Monitored markers were: MYOD, the earliest marker of myogenic commitment and MHY2, the gene coding for muscle myosin heavy chain 2.

**Fig. 5:**
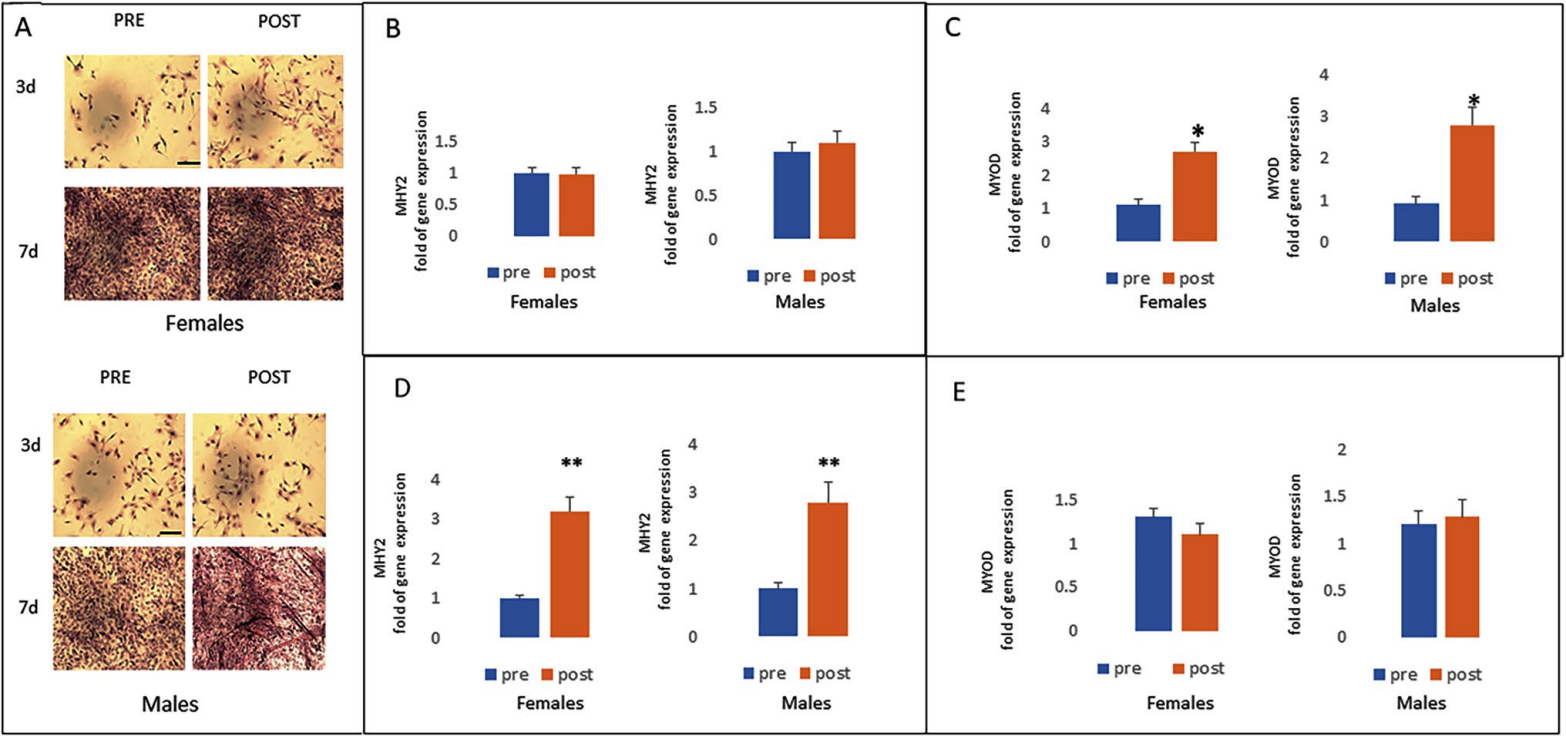
Ematoxilin stained myoblast cells cultured for 3 or 7 days with sera collected before (PRE) and after (POST) the half marathon in females (pre or post) or males (pre or post) (**A**). In both genders, after 3 days of cultivation the expression of MHY2 did not change (**B**) while MYOD gene expression increased in post HM sera conditioned-cells (**C**). The gene expression of MHY2 increased (**D**) while the expression of MYOD was not affected (**E**) after 7 days culture in the presence of post HM sera. scale bar 20 μm. Data are shown as mean ± standard deviation (SD); comparison was performed versus samples treated with pre HM in female or in male. *p < 0.05; **p < 0.005

Myoblast cells cultured for 3 days in the presence of sera collected before and after the half marathon (pHM, post Half Marathon) did not show any change in MHY2 expression in both genders, (Fig. 5B). However, we observed increased MYOD gene expression in pHM sera conditioned-cells (Fig. 5C). Expression of MHY2 gene increased (Fig. 5D) and the expression of MYOD decreased (Fig. 5E) after 7 days cultivation in the presence of pHM sera.

**Fig. 6:**
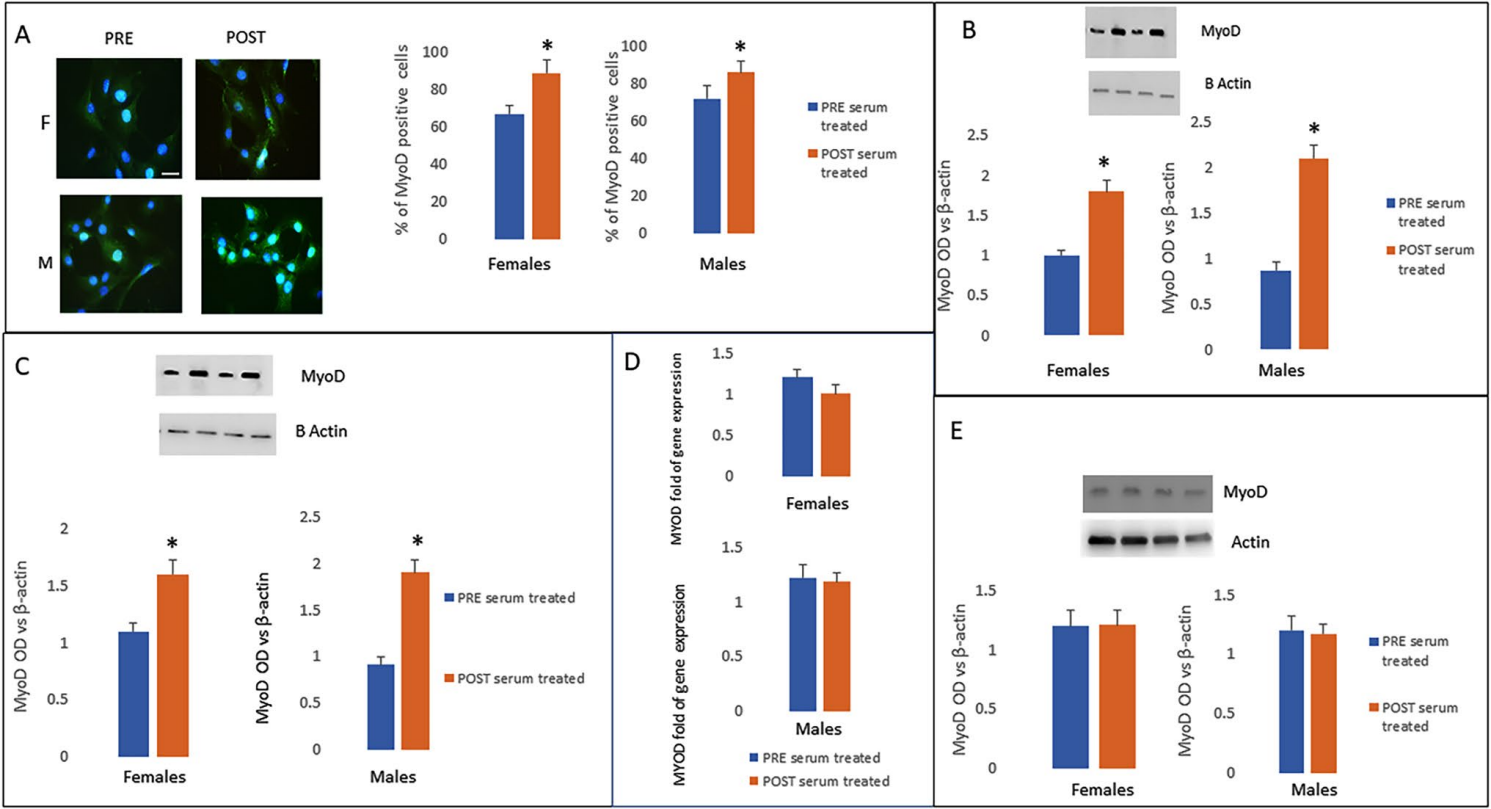
Increased number of MYOD positive cells (**A**) as well as MYOD protein levels (**B**) in both female and male post HM sera conditioned-cells was observed after 3 days of culture. MYOD protein levels were higher in cells cultured for 7 days in the presence of female or male post HM sera (**C**). After 14 days of culture no difference related to gene expression (**D**) or protein levels of MYOD (**E**) were observed in female or male post HM sera conditioned-cells. scale bar 50 μm. Data are shown as mean ± standard deviation (SD); comparison was performed versus samples treated with pre HM in female or in male. *p < 0.05

An increased number of MYOD positive cells (Fig. 6A) as well as increased MYOD protein levels (Fig. 6B) were observed in both female and male pHM sera conditioned-cells after 3 days of cultivation. However, we observed increased MYOD protein levels in both female and male pHM sera conditioned-cells also after 7 days (Fig. 6C).Furthermore, no differences in MYOD gene expression/protein levels were observed in both genders pHM sera conditioned-cells after 14 days of culture (Fig. 6D).

## Discussion

MicroRNAs (miRNAs or miRs) are short (∼22 nucleotides) single strand molecules which act as key regulators of a wide spectrum of cellular and physiological processes such as proliferation, differentiation, repair mechanisms, signaling and apoptosis. They can modulate the expression of target genes by binding to the 3′ untranslated region (3′UTR) of their transcripts, causing their degradation, or preventing the translational process [25]. These regulators are found in all cell types and also in extracellular vesicles, or associated to proteins that allow their transportation through extracellular fluids [26, 27]. Therefore, miRNAs are considered useful biomarkers in human health and disease [6, 28]. In particular, circulating miRNAs levels can be associated to exercise intensity and activity especially in relation to maximum oxygen uptake [29]. It has been suggested that exercise increases the number of Bone-Marrow-Derived Mesenchymal Stem Cells along with the enhancement of their osteogenic potential at the expense of the adipogenic commitment [30]; we recently demonstrated that physical activity promotes osteogenic differentiation by modulating different miRNAs expression [31]. However, in a previous study we did not observe different numbers of total progenitor cells but we found that osteogenic progenitors increased while adipogenic progenitors decreased after HM [22]. We analysed the effects of HM on the modulation of progenitor cells numerosity and differentiation trend immediately after HM; it might be interesting to analyse these cells after a longer interval from the physical performance.

Previous studies of miRNAs response to exercise have focused almost exclusively on male athletes. However, it is well known that females show gender-specific responses in cardiovascular [32], musculoskeletal [33] and metabolic [34] patterns following physical exercise. Sex-specific miRNAs modulation has been observed as a pathophysiological response to environmental stress [35]. Sex-specific miRNA patterns are thought to occur in response to gonadal steroids, such as dihydrotestosterone and progesterone. Notably, as gonadal steroids are affected during exercise, sex specific miRNAs expression levels have been considered in response to dihydrotestosterone and progesterone stimulation [36]. Therefore, in this study we aimed to analyse the gender role in miRNAs modulation following a half-marathon. In order to avoid any bias due to miRNAs profiles before the competition, we firstly evaluated eventual miRNAs expression differences between men and women. Interestingly, we observed similar miRNAs expressions, with rare exceptions. Mir223-3p and mir 26b levels appeared 17 and 14fold higher, respectively, in women compared to men. On the basis of our results, we hypothesized that miRNAs expression profiles observed in PBMCs before the competition might be reliable. In particular, the expression of some gender-related miRNAs is consistent with female physiology. Increased levels of mir223-3p in female PBMCs are in agreement with the role of this miRNA in endometrium, as suggested by Dong et al. [37]. In mice it has been demonstrated that mir223-3p interacts with LIF 3’UTR. In the female reproductive system, LIF (Leukemia Inhibitory Factor) is expressed in endometrial epithelial cells; its expression is higher in the luteal phase compared to proliferative phase [38, 39]. Therefore, the expression of mir223-3p correlates negatively with LIF levels in females. MiR-26b is abundantly expressed in the adipose tissue [40]. During adipogenic differentiation the expression of miR-26b is upregulated [41]. In addition, it has been suggested that the expression of miR 26b is directly related to the composition of milk fatty acids [41]. MiR-26b expression is also involved in the regulation of mammary epithelial cells together with lipid-related transcription factors in goat [40]. Therefore, our data suggest that differences in gender associated miRNAs modulation might be associated to female physiology and therefore to different hormonal statuses.

When we compared miRNA modulation in PBMCs following HM, we observed increased expression of miR152-3p, miR143-3p, miR27a-3p and reduced expression of miR30b in both women and men. However, the increased expression of several miRNAs such as miR22-3p and miR 100-5p was observed only in feminine PBMCs. Reduced levels of miR-22-3p have been observed in patients with premature ovarian failure [42]. Estrogen receptor 1 (ESR1) and phosphatase and tensin homolog (PTEN) have been indicated as candidate genes for ovarian failure [43]. In addition, by bioinformatic analyses miR-22-3p has been predicted to be a negative regulator of ESR1 and PTEN. Accordingly, higher miR100 -5p expression has been observed in granulosa cells of women with normal ovarian reserve compared to subjects with lower ovarian reserve [44]. Therefore, our data suggest a protective role of physical activity for the prevention of ovary dysfunctions.

Several miRNAs that were positively modulated following the half marathon in PBMCs from both genders, are supposed to be involved in skeletal muscle activity. MiR 143-3p expression is elevated in skeletal muscle tissue whereas its downregulation has been observed during aging [45]. It has been demonstrated that miR-143 acts as regulator of the insulin growth factor‐binding protein 5 (IGFBP-5) and that the expression of miR-143 and its target gene are disrupted in primary myoblasts affecting muscle regeneration [46]. It has been observed that miR-152 inhibits proliferation and promotes myoblast differentiation in C2C12 cell line [47]; in porcine myotube, the upregulation of miR-152 has been associated to the promotion of slow-twitch myofibers formation and myogenesis [48]. Accordingly, it has been demonstrated that miR-27a favors myoblasts proliferation by targeting the inhibitor of skeletal myogenesis Myostatin [49]. Recently, it has been demonstrated that miR‐23a and miR‐27a, both located in clusters, regulate proteins involved in the atrophy process. Interestingly, the authors showed that the overexpression of miR‐23a/27a in muscle is able to counteract the diabetes‐induced muscle cachexia [50]. As PBMCs have been validated as surrogate models for skeletal muscle tissue [51], we believe that miRNAs modulation observed in PBMCs may also occur in cells involved in myogenesis. To strengthen this hypothesis, we also evaluated the expression of circulating miRNAs and the possible modulation of ci-miRNAs following exercise. Our data demonstrate that ci-miRNAs reflect what was observed in PBMCs. In addition, we observed downregulation of circulating miRNA 200b-3p following HM and this variation was statistically significant. MiR 200b has been demonstrated to target the GATA transcription factor, which is involved in cardiac myocytes proliferation and differentiation [52].

To reinforce the observation of modulated myogenesis-related circulating miRNAs upon HM, we cultured the cells in the presence of sera collected from runners before and after the half marathon. Our results regarding MYOD (transcription factor for the myogenic commitment) expression, confirmed an increased myogenic differentiation in cells treated with sera collected after physical activity.

Therefore, despite the gender-associated differential expression of some miRNAs observed in PBMCs in physiological conditions, we can state that physical activity induces the modulation of muscle differentiation-associated miRNAs in both women and men.

## Supplementary Information

Below is the link to the electronic supplementary material.

ESM 1 (DOCX 28.6 KB)

ESM 2 (DOCX 28.5 KB)

ESM 3 (DOCX 26.4 KB)

ESM 4 (DOCX 26.8 KB)

## Data Availability

The datasets used and/or analysed during the current study are available from the corresponding author upon a reasonable request.
