# Supplementary material for: Physical Activity Modulates miRNAs Levels and Enhances MYOD Expression in Myoblasts
Source: Stem Cell Rev Rep. 2022 Mar 22;18(5):1865–74. doi: 10.1007/s12015-022-10361-9 (PMC9209351; doi:10.1007/s12015-022-10361-9)
Supplement: Supplementary file 1 — (DOCX 28.6 KB) [file 12015_2022_10361_MOESM1_ESM.docx]

Table 1S. Array expression in female before Half Marathon

|  |  |
| --- | --- |
|  | fold of expression |
| hsa-let-7a-5p-478575_mir | 5.76 |
| hsa-let-7b-5p-478576_mir | 0.06 |
| hsa-let-7d-5p-478439_mir | 1.06 |
| hsa-let-7e-5p-478579_mir | 0.24 |
| hsa-let-7f-5p-478578_mir | 4.73 |
| hsa-let-7g-5p-478580_mir | 3 |
| hsa-let-7i-5p-478375_mir | 6.57 |
| hsa-miR-101-3p-477863_mir | 2.09 |
| hsa-miR-103a-2-5p-477864_mir | 0.09 |
| hsa-miR-103a-3p-478253_mir | 1 |
| hsa-miR-106a-5p-478225_mir | 0.58 |
| hsa-miR-106b-5p-478412_mir | 1.53 |
| hsa-miR-107-478254_mir | 0.71 |
| hsa-miR-10a-5p-479241_mir | 0.03 |
| hsa-miR-125a-5p-477884_mir | 0.42 |
| hsa-miR-125b-5p-477885_mir | 0.14 |
| hsa-miR-126-3p-477887_mir | 4.81 |
| hsa-miR-127-3p-477889_mir | 0.04 |
| hsa-miR-128-3p-477892_mir | 0.24 |
| hsa-miR-130a-3p-477851_mir | 0.39 |
| hsa-miR-130b-3p-477840_mir | 0.08 |
| hsa-miR-132-3p-477900_mir | 0.03 |
| hsa-miR-133a-3p-478511_mir | 0.02 |
| hsa-miR-136-5p-478307_mir | 0.04 |
| hsa-miR-138-5p-477905_mir | 0.05 |
| hsa-miR-139-5p-478312_mir | 0.18 |
| hsa-miR-1-3p-477820_mir | 0.13 |
| hsa-miR-140-3p-477908_mir | 0.37 |
| hsa-miR-140-5p-477909_mir | 0.35 |
| hsa-miR-141-3p-478501_mir | 0.1 |
| hsa-miR-142-3p-477910_mir | 15.41 |
| hsa-miR-142-5p-477911_mir | 21.2 |
| hsa-miR-143-3p-477912_mir | 0.16 |
| hsa-miR-145-5p-477916_mir | 0.35 |
| hsa-miR-146a-5p-478399_mir | 3.07 |
| hsa-miR-146b-3p-478715_mir | 0.04 |
| hsa-miR-146b-5p-478513_mir | 2.72 |
| hsa-miR-148a-3p-477814_mir | 0.35 |
| hsa-miR-148b-3p-477824_mir | 0.45 |
| hsa-miR-150-5p-477918_mir | 67.02 |
| hsa-miR-152-3p-477921_mir | 0.11 |
| hsa-miR-155-5p-477927_mir | 0.34 |
| hsa-miR-15a-5p-477858_mir | 5.41 |
| hsa-miR-15b-5p-478313_mir | 18.17 |
| hsa-miR-16-5p-477860_mir | 9.91 |
| hsa-miR-16-5p-477860_mir | 8.87 |
| hsa-miR-16-5p-477860_mir | 8.28 |
| hsa-miR-16-5p-477860_mir | 7.87 |
| hsa-miR-16-5p-477860_mir | 7.27 |
| hsa-miR-17-5p-478447_mir | 4.66 |
| hsa-miR-181a-5p-477857_mir | 5.33 |
| hsa-miR-181b-5p-478583_mir | 0.69 |
| hsa-miR-181c-5p-477934_mir | 1.18 |
| hsa-miR-181d-5p-479517_mir | 0.13 |
| hsa-miR-182-5p-477935_mir | 0.26 |
| hsa-miR-183-5p-477937_mir | 0.23 |
| hsa-miR-185-5p-477939_mir | 0.44 |
| hsa-miR-186-5p-477940_mir | 0.96 |
| hsa-miR-18a-5p-478551_mir | 1.14 |
| hsa-miR-18b-5p-478584_mir | 0.31 |
| hsa-miR-190a-5p-478358_mir | 0.01 |
| hsa-miR-191-5p-477952_mir | 2.47 |
| hsa-miR-192-5p-478262_mir | 0.11 |
| hsa-miR-193a-5p-477954_mir | 0.01 |
| hsa-miR-193b-3p-478314_mir | 0.07 |
| hsa-miR-194-5p-477956_mir | 0.05 |
| hsa-miR-195-5p-477957_mir | 0.04 |
| hsa-miR-197-3p-477959_mir | 2.4 |
| hsa-miR-199a-3p_hsa-miR-199b-3p-477961_mir | 1.75 |
| hsa-miR-199a-5p-478231_mir | 0.24 |
| hsa-miR-199b-5p-478486_mir | 0.11 |
| hsa-miR-19a-3p-479228_mir | 2.29 |
| hsa-miR-19b-3p-478264_mir | 2.14 |
| hsa-miR-200a-3p-478490_mir | 0.01 |
| hsa-miR-200b-3p-477963_mir | 0.02 |
| hsa-miR-200c-3p-478351_mir | 0.05 |
| hsa-miR-20a-5p-478586_mir | 3.51 |
| hsa-miR-20b-5p-477804_mir | 0.45 |
| hsa-miR-210-3p-477970_mir | 0.05 |
| hsa-miR-2110-477971_mir | 0.02 |
| hsa-miR-2110-477971_mir | 0.02 |
| hsa-miR-212-3p-478318_mir | 0.02 |
| hsa-miR-212-3p-478318_mir | 0.02 |
| hsa-miR-215-5p-478516_mir | 0.01 |
| hsa-miR-21-5p-477975_mir | 15.08 |
| hsa-miR-21-5p-477975_mir | 15.08 |
| hsa-miR-217-478773_mir | 0.02 |
| hsa-miR-219a-5p-477980_mir | 0.01 |
| hsa-miR-219a-5p-477980_mir | 0.01 |
| hsa-miR-221-3p-477981_mir | 5.1 |
| hsa-miR-221-3p-477981_mir | 5.1 |
| hsa-miR-222-3p-477982_mir | 0.27 |
| hsa-miR-222-3p-477982_mir | 0.27 |
| hsa-miR-223-3p-477983_mir | 40.23 |
| hsa-miR-223-3p-477983_mir | 40.23 |
| hsa-miR-22-3p-477985_mir | 2.41 |
| hsa-miR-22-3p-477985_mir | 2.41 |
| hsa-miR-224-5p-477986_mir | 0.07 |
| hsa-miR-224-5p-477986_mir | 0.07 |
| hsa-miR-23a-3p-478532_mir | 9.63 |
| hsa-miR-23a-3p-478532_mir | 9.63 |
| hsa-miR-24-3p-477992_mir | 1.54 |
| hsa-miR-24-3p-477992_mir | 1.54 |
| hsa-miR-25-3p-477994_mir | 1.45 |
| hsa-miR-25-3p-477994_mir | 1.45 |
| hsa-miR-26a-5p-477995_mir | 15.74 |
| hsa-miR-26a-5p-477995_mir | 15.74 |
| hsa-miR-26b-5p-478418_mir | 31.35 |
| hsa-miR-26b-5p-478418_mir | 31.35 |
| hsa-miR-27a-3p-478384_mir | 7.73 |
| hsa-miR-27a-3p-478384_mir | 7.73 |
| hsa-miR-27b-3p-478270_mir | 2.27 |
| hsa-miR-27b-3p-478270_mir | 2.27 |
| hsa-miR-28-3p-477999_mir | 0.11 |
| hsa-miR-28-3p-477999_mir | 0.11 |
| hsa-miR-28-5p-478000_mir | 0.1 |
| hsa-miR-28-5p-478000_mir | 0.1 |
| hsa-miR-296-5p-477836_mir | 0.03 |
| hsa-miR-296-5p-477836_mir | 0.03 |
| hsa-miR-299-3p-478792_mir | 0.08 |
| hsa-miR-299-3p-478792_mir | 0.08 |
| hsa-miR-299-5p-478793_mir | 0.07 |
| hsa-miR-299-5p-478793_mir | 0.07 |
| hsa-miR-29a-3p-478587_mir | 5.3 |
| hsa-miR-29a-3p-478587_mir | 5.3 |
| hsa-miR-29b-3p-478369_mir | 5 |
| hsa-miR-29b-3p-478369_mir | 5 |
| hsa-miR-29c-3p-479229_mir | 4.39 |
| hsa-miR-29c-3p-479229_mir | 4.39 |
| hsa-miR-301a-3p-477815_mir | 0.13 |
| hsa-miR-301a-3p-477815_mir | 0.13 |
| hsa-miR-301b-3p-477825_mir | 0.03 |
| hsa-miR-301b-3p-477825_mir | 0.03 |
| hsa-miR-30b-5p-478007_mir | 2.62 |
| hsa-miR-30b-5p-478007_mir | 2.62 |
| hsa-miR-30c-5p-478008_mir | 1.79 |
| hsa-miR-30c-5p-478008_mir | 1.79 |
| hsa-miR-30e-5p-479235_mir | 0.81 |
| hsa-miR-30e-5p-479235_mir | 0.81 |
| hsa-miR-31-5p-478015_mir | 0.14 |
| hsa-miR-31-5p-478015_mir | 0.14 |
| hsa-miR-320a-478594_mir | 1.71 |
| hsa-miR-320a-478594_mir | 1.71 |
| hsa-miR-324-3p-478023_mir | 0.24 |
| hsa-miR-324-3p-478023_mir | 0.24 |
| hsa-miR-324-5p-478024_mir | 0.28 |
| hsa-miR-324-5p-478024_mir | 0.28 |
| hsa-miR-32-5p-478026_mir | 0.46 |
| hsa-miR-32-5p-478026_mir | 0.46 |
| hsa-miR-326-478027_mir | 0.31 |
| hsa-miR-326-478027_mir | 0.31 |
| hsa-miR-328-3p-478028_mir | 0.12 |
| hsa-miR-328-3p-478028_mir | 0.12 |
| hsa-miR-329-3p-478029_mir | 0.05 |
| hsa-miR-329-3p-478029_mir | 0.05 |
| hsa-miR-330-3p-478030_mir | 0.03 |
| hsa-miR-330-3p-478030_mir | 0.03 |
| hsa-miR-330-5p-478830_mir | 0.11 |
| hsa-miR-330-5p-478830_mir | 0.11 |
| hsa-miR-331-3p-478323_mir | 0.4 |
| hsa-miR-331-3p-478323_mir | 0.4 |
| hsa-miR-335-5p-478324_mir | 1.39 |
| hsa-miR-335-5p-478324_mir | 1.39 |
| hsa-miR-337-5p-478036_mir | 0.01 |
| hsa-miR-338-3p-478037_mir | 0.14 |
| hsa-miR-338-3p-478037_mir | 0.14 |
| hsa-miR-339-3p-478325_mir | 0.2 |
| hsa-miR-339-3p-478325_mir | 0.2 |
| hsa-miR-339-5p-478040_mir | 0.31 |
| hsa-miR-339-5p-478040_mir | 0.31 |
| hsa-miR-33b-5p-478479_mir | 0.07 |
| hsa-miR-33b-5p-478479_mir | 0.07 |
| hsa-miR-340-5p-478042_mir | 0.63 |
| hsa-miR-340-5p-478042_mir | 0.63 |
| hsa-miR-342-3p-478043_mir | 5.46 |
| hsa-miR-342-3p-478043_mir | 5.46 |
| hsa-miR-342-5p-478044_mir | 0.15 |
| hsa-miR-342-5p-478044_mir | 0.15 |
| hsa-miR-345-5p-478366_mir | 0.24 |
| hsa-miR-345-5p-478366_mir | 0.24 |
| hsa-miR-34a-5p-478048_mir | 0.04 |
| hsa-miR-34a-5p-478048_mir | 0.04 |
| hsa-miR-361-5p-478056_mir | 0.7 |
| hsa-miR-361-5p-478056_mir | 0.7 |
| hsa-miR-362-3p-478058_mir | 0.33 |
| hsa-miR-362-3p-478058_mir | 0.33 |
| hsa-miR-362-5p-478059_mir | 0.07 |
| hsa-miR-362-5p-478059_mir | 0.07 |
| hsa-miR-363-3p-478060_mir | 0.19 |
| hsa-miR-363-3p-478060_mir | 0.19 |
| hsa-miR-365a-3p_hsa-miR-365b-3p-478065_mir | 0.13 |
| hsa-miR-365a-3p_hsa-miR-365b-3p-478065_mir | 0.13 |
| hsa-miR-369-3p-478067_mir | 0.14 |
| hsa-miR-369-3p-478067_mir | 0.14 |
| hsa-miR-369-5p-478068_mir | 0.02 |
| hsa-miR-369-5p-478068_mir | 0.02 |
| hsa-miR-370-3p-478326_mir | 0.07 |
| hsa-miR-370-3p-478326_mir | 0.07 |
| hsa-miR-374a-5p-478238_mir | 0.81 |
| hsa-miR-374a-5p-478238_mir | 0.81 |
| hsa-miR-374b-5p-478389_mir | 0.45 |
| hsa-miR-374b-5p-478389_mir | 0.45 |
| hsa-miR-376a-3p-478240_mir | 0.06 |
| hsa-miR-376a-3p-478240_mir | 0.06 |
| hsa-miR-376c-3p-478459_mir | 0.16 |
| hsa-miR-376c-3p-478459_mir | 0.16 |
| hsa-miR-377-3p-478075_mir | 0.06 |
| hsa-miR-377-3p-478075_mir | 0.06 |
| hsa-miR-381-3p-477816_mir | 0.06 |
| hsa-miR-381-3p-477816_mir | 0.06 |
| hsa-miR-382-5p-478078_mir | 0.02 |
| hsa-miR-382-5p-478078_mir | 0.02 |
| hsa-miR-409-5p-478872_mir | 0.04 |
| hsa-miR-409-5p-478872_mir | 0.04 |
| hsa-miR-410-3p-478085_mir | 0.02 |
| hsa-miR-410-3p-478085_mir | 0.02 |
| hsa-miR-411-5p-478086_mir | 0.01 |
| hsa-miR-412-3p-478087_mir | 0.07 |
| hsa-miR-412-3p-478087_mir | 0.07 |
| hsa-miR-421-478088_mir | 0.05 |
| hsa-miR-421-478088_mir | 0.05 |
| hsa-miR-423-3p-478327_mir | 1.1 |
| hsa-miR-423-3p-478327_mir | 1.1 |
| hsa-miR-423-5p-478090_mir | 1.63 |
| hsa-miR-423-5p-478090_mir | 1.63 |
| hsa-miR-424-5p-478092_mir | 0.18 |
| hsa-miR-424-5p-478092_mir | 0.18 |
| hsa-miR-425-5p-478094_mir | 0.58 |
| hsa-miR-425-5p-478094_mir | 0.58 |
| hsa-miR-431-5p-478889_mir | 0.08 |
| hsa-miR-431-5p-478889_mir | 0.08 |
| hsa-miR-433-3p-478102_mir | 0.02 |
| hsa-miR-433-3p-478102_mir | 0.02 |
| hsa-miR-450a-5p-478106_mir | 0.11 |
| hsa-miR-450a-5p-478106_mir | 0.11 |
| hsa-miR-451a-478107_mir | 1.92 |
| hsa-miR-451a-478107_mir | 1.92 |
| hsa-miR-454-3p-478329_mir | 0.42 |
| hsa-miR-454-3p-478329_mir | 0.42 |
| hsa-miR-484-478308_mir | 1.03 |
| hsa-miR-484-478308_mir | 1.03 |
| hsa-miR-485-3p-478125_mir | 0.05 |
| hsa-miR-485-3p-478125_mir | 0.05 |
| hsa-miR-486-3p-478422_mir | 0.02 |
| hsa-miR-486-3p-478422_mir | 0.02 |
| hsa-miR-486-5p-478128_mir | 0.16 |
| hsa-miR-486-5p-478128_mir | 0.16 |
| hsa-miR-487a-3p-477826_mir | 0.08 |
| hsa-miR-487a-3p-477826_mir | 0.08 |
| hsa-miR-487b-3p-477835_mir | 0.08 |
| hsa-miR-487b-3p-477835_mir | 0.08 |
| hsa-miR-490-3p-478131_mir | 0.03 |
| hsa-miR-490-3p-478131_mir | 0.03 |
| hsa-miR-491-3p-478942_mir | 0.08 |
| hsa-miR-491-3p-478942_mir | 0.08 |
| hsa-miR-491-5p-478132_mir | 0.01 |
| hsa-miR-494-3p-478135_mir | 0.06 |
| hsa-miR-494-3p-478135_mir | 0.06 |
| hsa-miR-495-3p-478136_mir | 0.14 |
| hsa-miR-495-3p-478136_mir | 0.14 |
| hsa-miR-496-478335_mir | 0.05 |
| hsa-miR-496-478335_mir | 0.05 |
| hsa-miR-500a-5p-478309_mir | 0.03 |
| hsa-miR-500a-5p-478309_mir | 0.03 |
| hsa-miR-502-3p-478348_mir | 0.04 |
| hsa-miR-502-3p-478348_mir | 0.04 |
| hsa-miR-502-5p-478954_mir | 0.02 |
| hsa-miR-502-5p-478954_mir | 0.02 |
| hsa-miR-503-5p-478143_mir | 0.01 |
| hsa-miR-505-3p-478145_mir | 0.11 |
| hsa-miR-505-3p-478145_mir | 0.11 |
| hsa-miR-507-478960_mir | 0.02 |
| hsa-miR-507-478960_mir | 0.02 |
| hsa-miR-515-5p-478147_mir | 0.02 |
| hsa-miR-515-5p-478147_mir | 0.02 |
| hsa-miR-532-3p-478336_mir | 0.09 |
| hsa-miR-532-3p-478336_mir | 0.09 |
| hsa-miR-532-5p-478151_mir | 0.06 |
| hsa-miR-532-5p-478151_mir | 0.06 |
| hsa-miR-541-3p-478999_mir | 0.16 |
| hsa-miR-542-3p-478153_mir | 0.02 |
| hsa-miR-542-3p-478153_mir | 0.02 |
| hsa-miR-544a-478156_mir | 0.03 |
| hsa-miR-544a-478156_mir | 0.03 |
| hsa-miR-545-3p-479002_mir | 0.04 |
| hsa-miR-545-3p-479002_mir | 0.04 |
| hsa-miR-548a-3p-478157_mir | 0.01 |
| hsa-miR-548a-3p-478157_mir | 0.01 |
| hsa-miR-548b-3p-479018_mir | 0.02 |
| hsa-miR-548b-3p-479018_mir | 0.02 |
| hsa-miR-551b-3p-478159_mir | 0.06 |
| hsa-miR-551b-3p-478159_mir | 0.06 |
| hsa-miR-574-3p-478163_mir | 0.13 |
| hsa-miR-574-3p-478163_mir | 0.13 |
| hsa-miR-576-5p-478165_mir | 0.05 |
| hsa-miR-576-5p-478165_mir | 0.05 |
| hsa-miR-579-3p-479059_mir | 0.07 |
| hsa-miR-579-3p-479059_mir | 0.07 |
| hsa-miR-582-5p-478166_mir | 0.03 |
| hsa-miR-582-5p-478166_mir | 0.03 |
| hsa-miR-590-5p-478367_mir | 0.1 |
| hsa-miR-590-5p-478367_mir | 0.1 |
| hsa-miR-597-5p-478339_mir | 0.03 |
| hsa-miR-597-5p-478339_mir | 0.03 |
| hsa-miR-598-3p-478172_mir | 0.02 |
| hsa-miR-598-3p-478172_mir | 0.02 |
| hsa-miR-615-5p-478176_mir | 0.04 |
| hsa-miR-625-5p-479469_mir | 0.21 |
| hsa-miR-625-5p-479469_mir | 0.21 |
| hsa-miR-628-5p-479112_mir | 0.02 |
| hsa-miR-636-478185_mir | 0.03 |
| hsa-miR-636-478185_mir | 0.03 |
| hsa-miR-651-5p-479131_mir | 0.12 |
| hsa-miR-651-5p-479131_mir | 0.12 |
| hsa-miR-652-3p-478189_mir | 1.63 |
| hsa-miR-652-3p-478189_mir | 1.63 |
| hsa-miR-654-3p-479135_mir | 0.31 |
| hsa-miR-654-3p-479135_mir | 0.31 |
| hsa-miR-654-5p-478368_mir | 0.02 |
| hsa-miR-654-5p-478368_mir | 0.02 |
| hsa-miR-655-3p-478191_mir | 0.01 |
| hsa-miR-660-5p-478192_mir | 0.06 |
| hsa-miR-660-5p-478192_mir | 0.06 |
| hsa-miR-671-3p-478194_mir | 0.06 |
| hsa-miR-671-3p-478194_mir | 0.06 |
| hsa-miR-744-5p-478200_mir | 0.34 |
| hsa-miR-744-5p-478200_mir | 0.34 |
| hsa-miR-758-3p-479166_mir | 0.03 |
| hsa-miR-758-3p-479166_mir | 0.03 |
| hsa-miR-873-5p-478204_mir | 0.01 |
| hsa-miR-874-3p-478205_mir | 0.04 |
| hsa-miR-874-3p-478205_mir | 0.04 |
| hsa-miR-876-5p-479187_mir | 0.03 |
| hsa-miR-876-5p-479187_mir | 0.03 |
| hsa-miR-889-3p-478208_mir | 0.12 |
| hsa-miR-889-3p-478208_mir | 0.12 |
| hsa-miR-92a-3p-477827_mir | 7.42 |
| hsa-miR-92a-3p-477827_mir | 7.42 |
| hsa-miR-92b-3p-477823_mir | 8.11 |
| hsa-miR-92b-3p-477823_mir | 8.11 |
| hsa-miR-93-5p-478210_mir | 0.58 |
| hsa-miR-93-5p-478210_mir | 0.58 |
| hsa-miR-9-5p-478214_mir | 0.01 |
| hsa-miR-98-5p-478590_mir | 0.26 |
| hsa-miR-99a-5p-478519_mir | 0.22 |
| hsa-miR-99a-5p-478519_mir | 0.22 |
| hsa-miR-99b-5p-478343_mir | 0.08 |
| hsa-miR-99b-5p-478343_mir | 0.08 |
